# Feedback Type May Change the EMG Pattern and Kinematics During Robot Supported Upper Limb Reaching Task

**DOI:** 10.1109/OJEMB.2024.3363137

**Published:** 2024-02-07

**Authors:** Yasuhiro Kato, Toshiaki Tsuji, Imre Cikajlo

**Affiliations:** ^1^ Graduate School of Science and EngineeringSaitama University 13032 Sakura-ku 338-8570 Japan; ^2^ University Rehabilitation Institute Republic of Slovenia 112781 1000 Ljubljana Slovenia; ^3^ School of Engineering and ManagementUniversity of Nova Gorica 119110 5271 Vipava Slovenia

**Keywords:** Movement strategy, multimodal feedback, sEMG analysis

## Abstract

Haptic interfaces and virtual reality (VR) technology have been increasingly introduced in rehabilitation, facilitating the provision of various feedback and task conditions. However, correspondence between the feedback/task conditions and movement strategy during reaching tasks remains a question. To investigate movement strategy, we assessed velocity parameters and peak latency of electromyography. Ten neuromuscularly intact volunteers participated in the measurement using haptic interface and VR. Concurrent visual feedback and various terminal feedback (e.g., visual, haptic, visual and haptic) were given. Additionally, the object size for the reaching task was changed. The results demonstrated terminal haptic feedback had a significant impact on kinematic parameters; showed $0.7\,\pm {\,1.4}$ s ($p\,< .05$) shorter movement time and $0.01\,\pm {\,0.08}$ m/s ($p\,< .05$) higher mean velocity compared to no terminal feedback. Also, smaller peak latency was observed in different muscle regions based on the object size.

## I. Introduction

**Figure 1.**
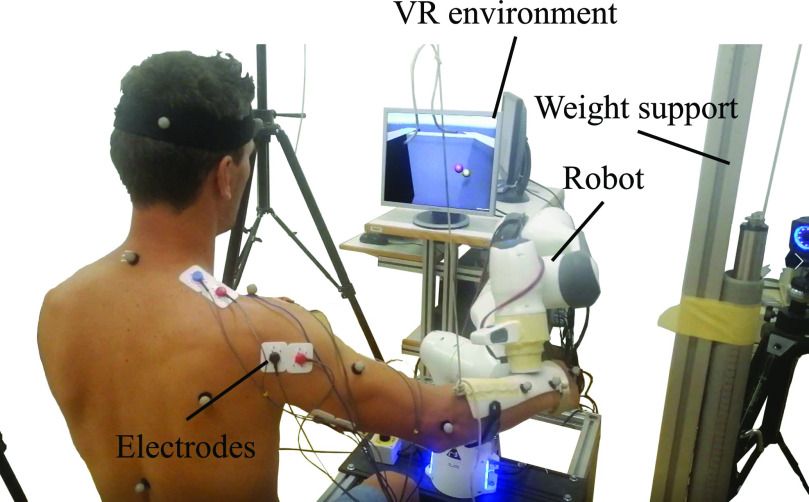
Robot-supported upper-limb reaching task: The 3-dimensional upper limb reaching exercise consisted of the VR environment and the robot. Human users can interact with VR objects using the robot as a haptic interface. Haptic sensations of the VR objects are provided by the robot.

As robotic technology has developed, it has been introduced into motor learning and rehabilitation [1], [2], [3], [4]. The benefits of using robots include objective evaluation through sensing technology, the ability to continuously execute tasks, and the provision of feedback or guidance through various modalities (see Fig. 1). Providing feedback is often incorporated in motor learning, and numerous studies have been conducted to understand the influence of feedback modalities, such as visual [5], [6], auditory [7], and haptic [8], [9], [10], [11], [12], [13], [14], [15], [16], [17], [18], [19], [20]. However, for a deeper understanding of the kinematic influence of the feedback, various parameters should be considered and selected.

Kinematic measurement is a reliable predictor of psychomotor task (e.g., 3-dimensional goal-directed movements) in VR simulation as reported in [21]. Neta et al. investigated the training effect on stroke patients using a target-to-target task performance framework [22]. In this study, various velocity parameters were used to assess their influence on kinematics. They reported that the difference in task performance between healthy participants and stroke patients with respect to velocity profiles was observable, demonstrating the sensitivity of these parameters. However, the influence of feedback modalities was beyond the scope of the study.

Another approach for understanding the influence of feedback is to observe muscle activities by assessing electromyography (EMG) signals. Afzaf et al. used kinesthetic and vibrotactile feedback for gait training and reported that this feedback influenced the increase in gait speed, which was captured by EMG peak value observation [23]. In addition, Cikajlo et al. investigated the correlation between feedback modalities and EMG latency in a gait balancing task [24]. However, it has not been adequately demonstrated whether such an index, i.e., EMG latency, can be used to differentiate between the influences dependent on feedback modalities in a rather dynamic task. Based on the aforementioned studies, we addressed the following questions: (1) How do different feedback modalities influence kinematics in the reaching task framework? (2) Can EMG latency capture the influence of feedback modalities on kinematics during a dynamic task?

In this study, we examined how various types of feedback influence the reaching task strategy, which is described by kinematics and muscle activity patterns under various conditions. Thus far, the task was modified; the goal remained the same, but the size of the object was different. Given that previous studies have suggested changes in task performance or movement control [25] based on size-related properties such as the size-ratio between virtual and real-world [26], [27] or familiarity [28], we posited that the impact of feedback might also be influenced by object size. In order to evaluate the reaching task strategy, we developed a 3-dimensional reaching exercise in a VR space by using a robot. The setup incorporated VR and a cooperative robot as a haptic interface, which enabled us to provide multimodal feedback, including visual and haptic feedback, during the reaching exercise. Apart from feedback modalities, we also designed feedback delivery methods, such as concurrent feedback which is continuously presented, and terminal feedback presented when the task is completed, based on the feedback purpose.

We evaluated the reaching task strategy in the upper limb (UL) reaching task with respect to muscle activity patterns (e.g., peak latency) and velocity profiles (e.g., movement time, mean velocity, mean peak velocity, mean time to peak, and mean velocity peak number).

## II. Results

### A. Movement Time

**Figure 2.**
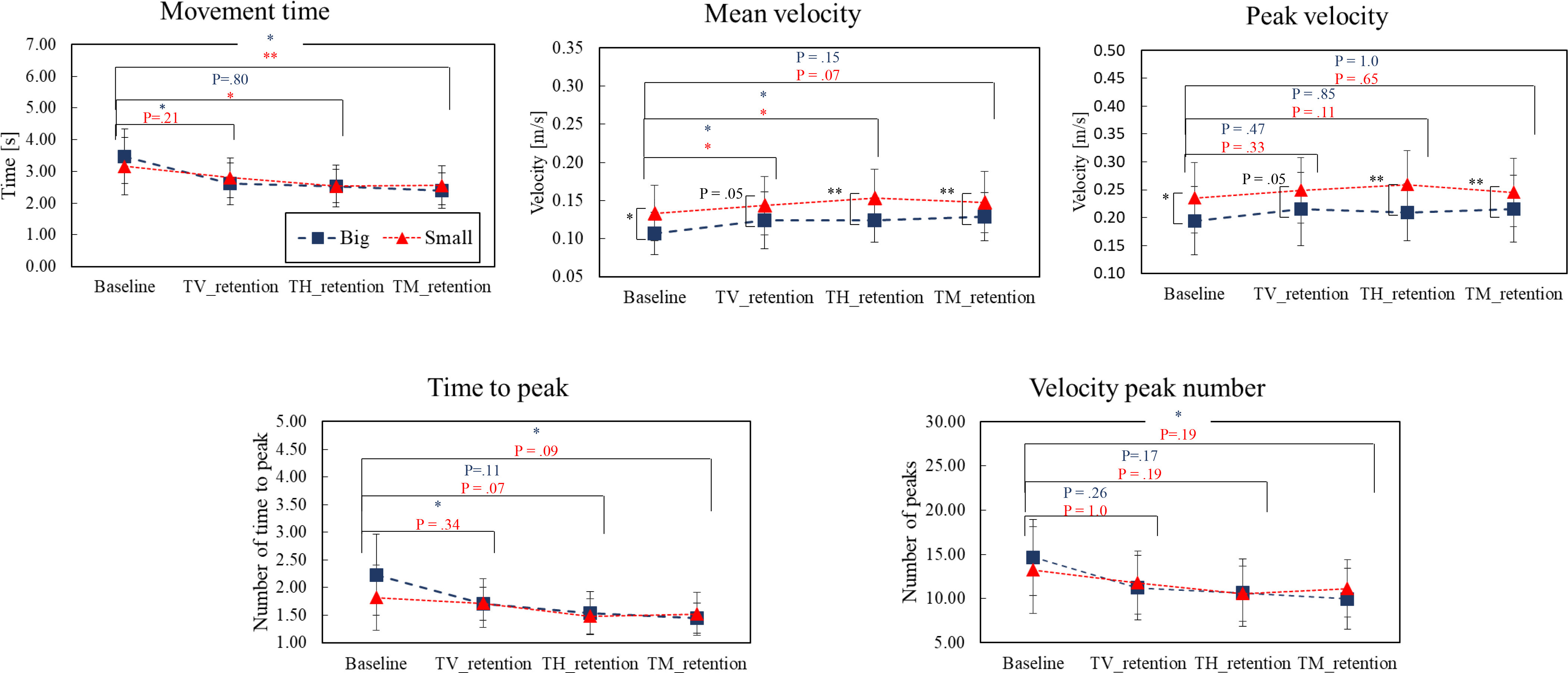
Results of the velocity profiles for 10 participants during the 3-dimensional UL reaching task. Blue dots represented big-ball mode while red dots represented small-ball mode. It was compared with each of the velocity parameters based on feedback modalities (TV: terminal visual feedback, TH: terminal haptic feedback, and TM: terminal multi-feedback). The results showed that there were significant differences, especially in movement time, mean speed, and mean time to peak. Asterisks indicated the level of statistical significance after post-hoc tests: $^{\ast } \mathit{p} < .05$ and $^{\ast \ast }\mathit{p} < .01$.

As shown in Fig. 2, the results indicated that haptic and multi-feedback modalities result in shorter movement times. In the big-ball mode, the baseline movement time was $t\,=\,3.5$ s, whereas terminal visual feedback TV: $t\,=\,2.6$ s, terminal haptic feedback TH: $t\,=\,2.5$ s, and terminal multi feedback TM: $t\,=\,2.4$ s. Thus, TH and TM resulted in shorter movement times compared to TV. This trend became even more prominent in the small-ball mode. In the small-ball mode, the baseline movement time was $t\,=\,3.2$ s, whereas TV: $t\,=\,2.8$ s, TH: $t\,=\,2.5$ s, and TM: $t\,=\,2.5$ s. Feedback had a significant effect according to the result in Table 1. Statistical significance was obtained in most cases (feedback modalities and ball size) when comparing the baseline with each retention.

**TABLE 1:** P Values of Velocity Profiles

Kinematics parameter	Ballsize	Feedback	Ballsize + feedback
Movement time	0.903	$1.3 \times 10^{-5***}$	$0.013^{*}$
Mean velocity	$0.002^{**}$	$1.9 \times 10^{-5***}$	0.445
Peak velocity	$0.003^{**}$	0.066	0.454
Time to peak	0.240	$2.6 \times 10^{-4***}$	$0.016^{*}$
Velocity peak number	0.916	$1.4 \times 10^{-4***}$	$0.015^{*}$

Statistical significance: $^{\ast } \mathit{p} < .05$, $^{\ast \ast }\mathit{p} < .01$, and $^{\ast \ast \ast }\mathit{p} < .001$.

### B. Mean Velocity and Peak Velocity

The mean velocity, $\mathit{v}_{\text{avg}}$ of each movement segment was calculated to evaluate the velocity profile in 3-dimensional Euclidean space during the reaching task. In the big-ball mode, the baseline mean velocity was $\mathit{v}_{\text{avg}}$ = 0.11 m/s, while TV: $\mathit{v}_{\text{avg}}$ = 0.12 m/s, TH: $\mathit{v}_{\text{avg}}$ = 0.12 m/s, and TM: $\mathit{v}_{\text{avg}}$ = 0.13 m/s. In the small-ball mode, the baseline mean velocity was $\mathit{v}_{\text{avg}}$ = 0.13 m/s, whereas TV: $\mathit{v}_{\text{avg}}$ = 0.14 m/s, TH: $\mathit{v}_{\text{avg}}$ = 0.15 m/s, and TM: $\mathit{v}_{\text{avg}}$ = 0.15 m/s. In both modes, the mean velocity rather increased in haptic/multi-feedback.

In terms of peak velocity $\mathit{v}_{\text{peak}}$, the baseline peak velocity in the big-ball mode was $\mathit{v}_{\text{peak}}$ = 0.19 m/s and TV: $\mathit{v}_{\text{peak}}$ = 0.22 m/s, TH: $\mathit{v}_{\text{peak}}$ = 0.21 m/s, and TM: $\mathit{v}_{\text{peak}}$ = 0.22 m/s. In the small-ball mode, the baseline peak velocity was $\mathit{v}_{\text{peak}}$ = 0.24 m/s, while TV: $\mathit{v}_{\text{peak}}$ = 0.25 m/s, TH: $\mathit{v}_{\text{peak}}$ = 0.26 m/s and TM: $\mathit{v}_{\text{peak}}$ = 0.25 m/s. Like the mean velocity, the peak velocity tended to increase with haptic feedback and multi-feedback.

### C. Velocity Peak Number and Time to Peak

The velocity peak number $\mathit{N}_{\text{peak}}$ was measured as an index of movement smoothness during the reaching task. In the big-ball mode, the baseline velocity peak number was $\mathit{N}_{\text{peak}}$ = 14.6, TV: $\mathit{N}_{\text{peak}}$ = 11.2, TH: $\mathit{N}_{\text{peak}}$ = 10.7, and TM: $\mathit{N}_{\text{peak}}$ = 10. In small-ball mode, the baseline velocity peak number was $\mathit{N}_{\text{peak}}$ = 13.3, whereas TV: $\mathit{N}_{\text{peak}}$ = 11.8, TH: $\mathit{N}_{\text{peak}}$ = 10.6, and TM: $\mathit{N}_{\text{peak}}$ = 11.1. As a result, regardless of the feedback modalities and task conditions, the reaching movement became smoother than the baseline.

The time to peak divides the movement profile as “ballistic phase” and “correction phase” and thus, this parameter indicates how participants changed their movement strategies during the task. As shown in Fig. 2, the baseline time to peak in big ball-mode was $t\,=\,2.2$ s and TV: $t\,=\,1.7$ s, TH: $t\,=\,1.5$ s and TM: $t\,=\,1.5$ s. In small-ball mode, the baseline time to peak was $t\,=\,1.8$ s, while TV: $t\,=\,1.7$ s, TH: $t\,=\,1.5$ s, and TM: $t\,=\,1.5$ s. Using the feedback with all three modalities showed a shorter time to peak. Rather, haptic and multi-feedback showed a shorter time to peak compared to visual feedback.

### D. sEMG Analysis

**Figure 3.**
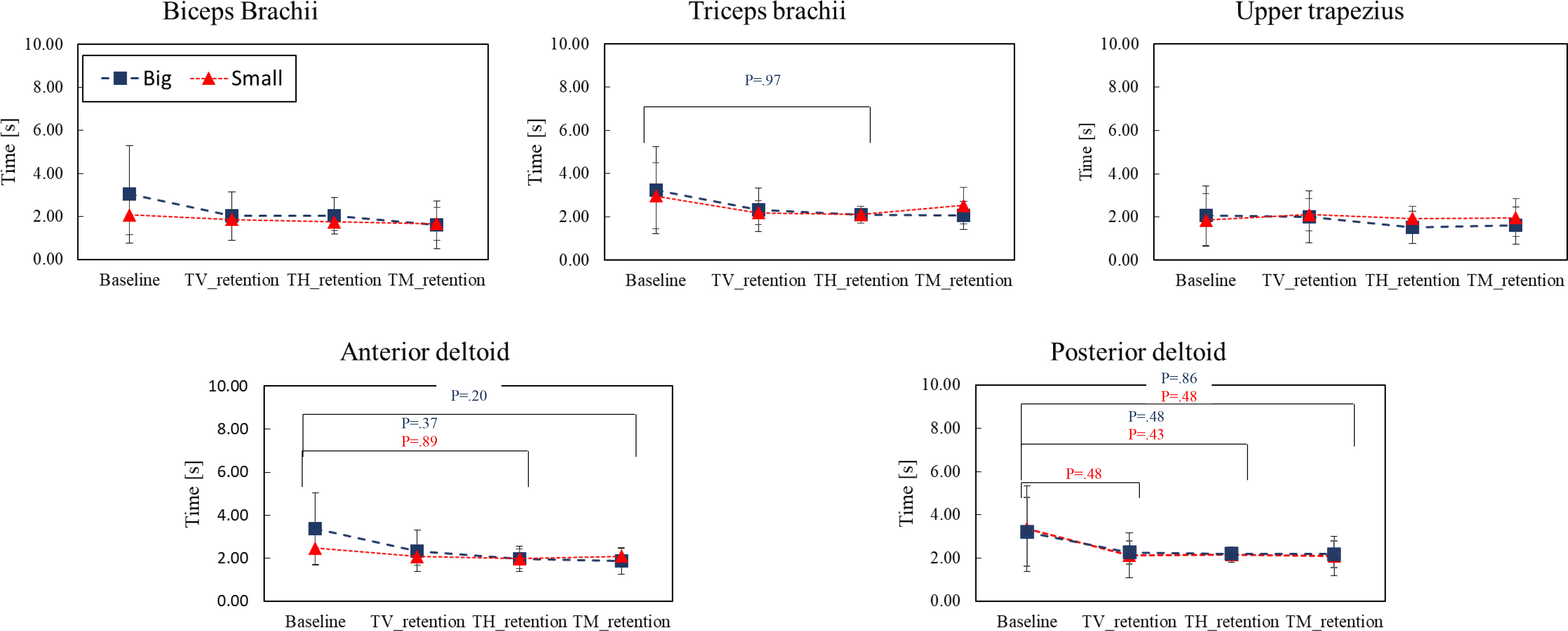
Results of the sEMG peak latency for ten participants during the 3-dimensional UL reaching task. Likewise, in the results of velocity profiles of Fig. 2, blue dots represented big-ball mode while red dots represented small-ball mode. The velocity parameters were compared based on feedback modalities (TV: terminal visual feedback, TH: terminal haptic feedback, and TM: terminal multi-modal feedback). It was observed that the biceps brachii had a smaller peak latency in the small-ball mode while the upper trapezius had a smaller peak latency in the big-ball mode.

The results of sEMG peak latency analysis were shown in Fig. 3. Peak latency is the interval between when peak sEMG is observed after target ball presentation. The main effect and interaction effect of ballsize and feedback modalities were shown in the Table 2. It was found that in the big-ball mode, the muscle activity of upper trapezius reached its peak value first (e.g., TV: $t\,=\,2.0$ s, TH: $t\,=\,1.5$ s, TM: $t\,=\,1.6$ s). On the other hand, in the small-ball task, the peak latency value of biceps brachii was smaller (e.g., TV: $t\,=\,1.9$ s, TH: $t\,=\,1.8$ s, TM: $t\,=\,1.7$ s). Thus, it was observed that muscle activation patterns were different based on the object size.

**TABLE 2:** P Values of sEMG Peak Latency

Muscles	Ballsize	Feedback	Ballsize + feedback
Biceps brachii	0.329	0.144	0.662
Triceps brachii	0.940	0.050	0.433
Upper Trapezius	0.424	0.717	0.727
Anterior deltiod	0.191	$1.0 \times 10^{-4***}$	0.346
Posterior deltoid	0.810	$0.032^{*}$	0.979

Statistical significance: $^{\ast } \mathit{p} < .05$, and $^{\ast \ast \ast }\mathit{p} < .001$.

## III. Discussion

This study investigated the effects of different feedback types on movement strategies during reaching tasks in a virtual reality environment. In addition to concurrent visual feedback, terminal feedback using three different modalities (visual, haptic, and multimodal) was used for the reaching task. Kinematic and sEMG data were analyzed under two conditions: big-ball mode and small-ball mode, which required different reaching distances due to the change in target size.

Terminal haptic feedback was found to have a larger impact on velocity profiles in time-independent reaching tasks in 3D space compared to other terminal feedback modalities (visual, multimodal). It resulted in shorter movement times and time to peak, as well as fewer peak velocities in both big-ball and small-ball modes. In the small-ball mode, mean and peak velocities were higher with the terminal haptic feedback modalities. Functional refinement in daily activities when employing terminal haptic feedback may be attributed to alterations in the magnitude of changes in movement time [29]. Although multimodal feedback is expected to be more effective for motor learning due to its enriched information, this study did not confirm the significant superiority of terminal multimodal feedback over haptic feedback. This could be due to an increase in cognitive load, as reducing attention demand enhances skill learning [30].

Concurrent visual feedback displayed the target position and the real-time hand position of the participants. Terminal feedback was used to indicate the task result, namely whether the participants reached the target or not. This feedback is considered knowledge of the results. Visual terminal feedback was designed to change the target color when it was reached. The terminal haptic feedback was designed to convey the sensation of a spherical shape when it was reached. Our study found that the velocity profile between baseline and retention changed significantly when using the visual terminal feedback we designed. However, our results suggested that terminal haptic feedback may be more intuitive to human cognition, as we observed larger differences between baseline and retention when using haptic feedback. In addition, tactile information about the virtual object was expected to be a useful form of feedback in a task where the goal is to reach and touch the object. These findings are consistent with previous research by Ebied et al., which reported that cutaneous sensations affect movement duration and jerk in handwriting tasks [31].

Haptic interaction has a significant impact on motor learning and development processes [32], [33]. In the 3-dimensional reaching task performed in our study, spatial recognition was crucial, and haptic feedback contributed more to a higher awareness of contact, which enabled participants to execute the task accurately [34], [35]. Based on our results, we assumed that terminal haptic feedback had a greater impact on the velocity profiles of healthy participants' movements in the 3-dimensional reaching task framework. Furthermore, we demonstrated the sensitivity of velocity parameters used in this study, indicating that these parameters could be indicators of movement strategy changes.

We focused on sEMG peak latency as an index to capture changes in activation patterns in the acquired signals. The sEMG peak latency is expected to correlate more with information about motion, such as velocity, than with information about human dynamics. In this study, we took the approach of evaluating changes in movement strategy from a kinematic perspective. Therefore, peak latency was chosen to determine how muscle activation patterns were influenced by various types of feedback. The results did not show significant differences in the peak latency of sEMG signals based on feedback modality. However, the results revealed that in the big-ball mode, the peak latency of the sEMG was smaller at upper trapezius. In contrast, in the small-ball task, the peak latency value of the biceps brachii was smaller. In other words, this may suggest that the UL movement strategy has changed due to the target size change, even though the position of the target remains the same. This finding would be useful for designing rehabilitation exercises in VR environments with haptic interfaces or for robot motion planning in rehabilitation frameworks.

## IV. Conclusion

This study investigated how feedback modalities influence the movement strategy during reaching tasks. For this purpose, various velocity parameters and sEMG peak latency were used to evaluate the influence on movement strategy. The results showed that terminal haptic feedback has highly changed the velocity parameters, compared to other terminal feedbacks (visual, multimodal). In addition, the sEMG analysis demonstrated that muscle activity patterns were different based on the object size in VR. Therefore, it was suggested that humans change their movement strategy based on the object size. In the future, measurements of participants following a stroke with the current experimental setup should be conducted to compare the influence of various feedback-to-movement strategies in healthy participants and stroke populations.

## V. Measurement

The setup of the measurement is based on our preliminary study [36]. The main components of the setup include the FRANKA EMIKA Panda robot arm (robot), a VR environment created using Unity 3D, and a Noraxon system for sEMG recording. The robot served as both a haptic interface and for kinematics recording. Visual information, including the VR, was presented through a 19-inch screen. In this section, we explain the feedback design of the system, the analysis methods, and the exercise protocol.

### A. VR Scene Design

**Figure 4.**
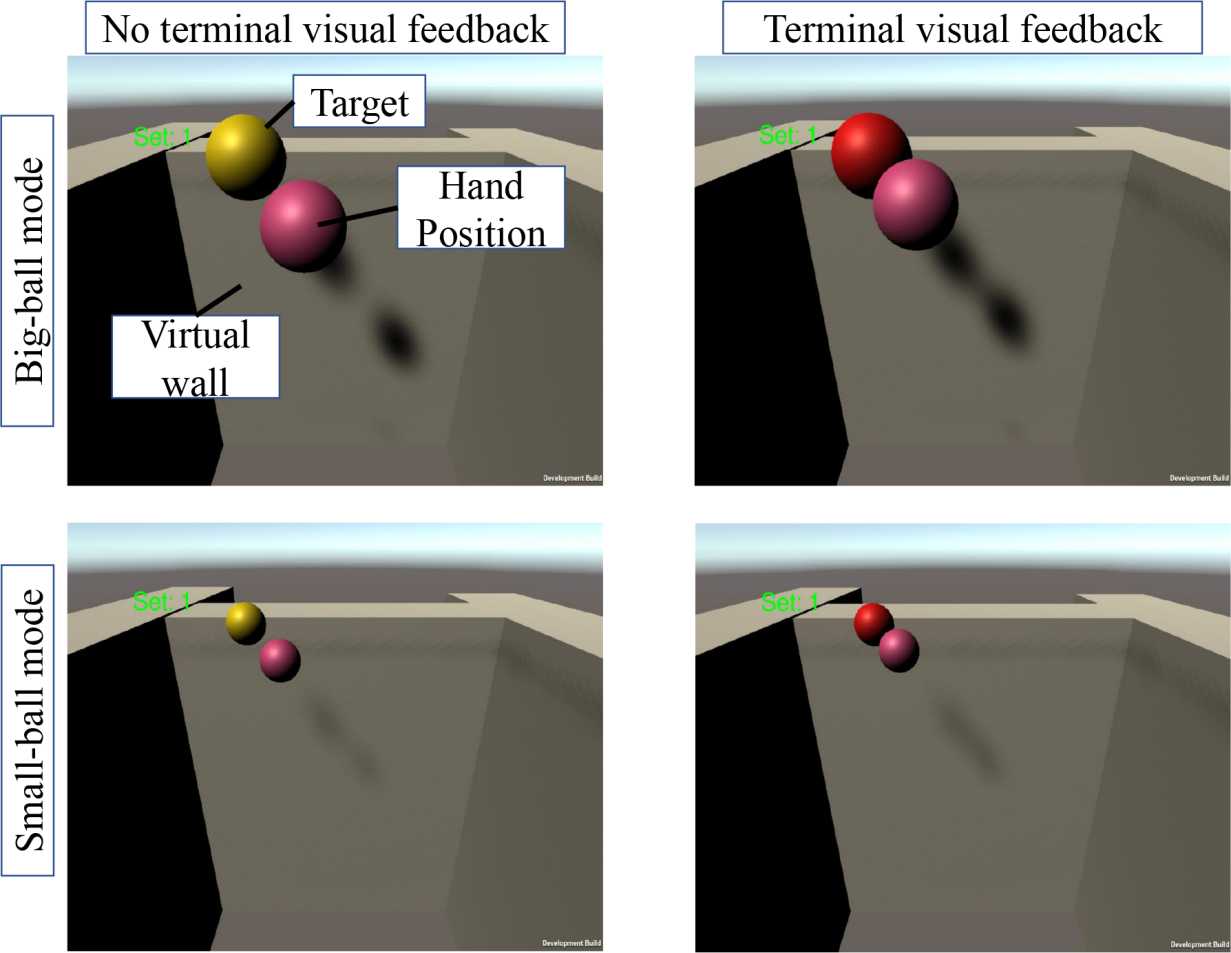
VR environment illustration: two balls were displayed which were surrounded by the virtual wall on the screen. The yellow ball represented the target position to which the users were required to reach their arms. The pink ball was the current hand position of the users. The virtual wall defined the workspace of the reaching task. It can prevent users from overreaching motions by providing haptic feedback. The top figures are big-ball mode and the bottom figures are small-ball mode which required a longer reaching motion than big-ball mode. The figures also illustrate terminal visual feedback. The target ball turned from yellow to red during the contact with the pink-colored ball.

**Figure 5.**
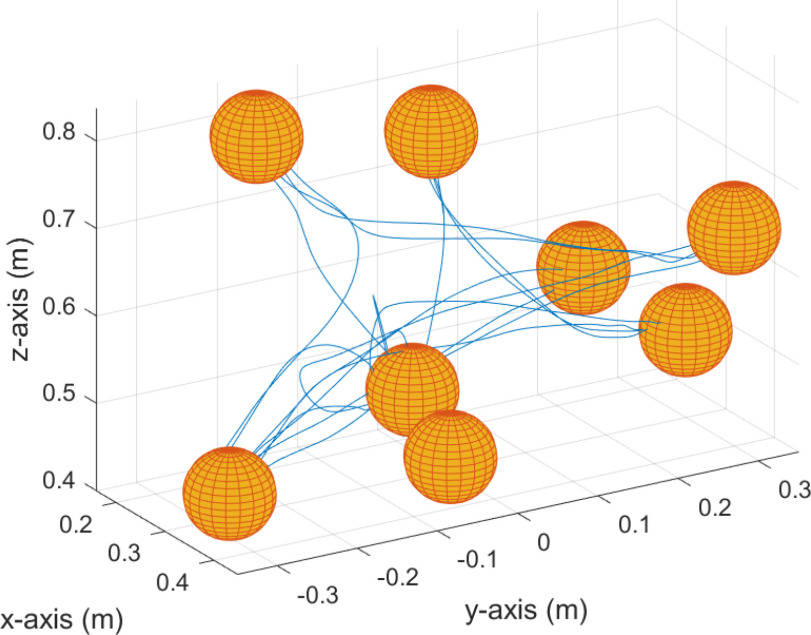
Sample reaching motion of the representative participant. The yellow ball is the target object and the blue line is the reaching trajectory.

Visual feedback was designed based on the insights of [5], [11], [19]. We designed a 3-dimensional reaching task for the UL in the VR environment. In the VR environment, two balls were displayed on a screen as shown in Fig. 4. The yellow ball represented the target object of the reaching task, whereas the pink ball indicated the hand position of the user. In the reaching task, the target positions were predetermined at eight locations as shown in Fig. 5. There were approximately $5.6\,\pm {3.4}$ cm of movement distance in small-ball mode, while the distance between targets in big-ball mode was $3.9\,\pm {3.7}$ cm. The initial position of the target object was fixed, while the rest of the target placements were randomized to prevent participants from learning the reaching trajectory. The target object changed position after contact with the ball representing the user's hand. The ball representing the user's hand was displaced and corresponded to the end-effector motion of the robot in real-time. Additionally, the size of the ball representing the target object could be adjusted to ‘big-ball mode’ and ‘small-ball mode’. The ball size adjustment allowed us to change the reaching distance. Moreover, the workspace was surrounded by a virtual wall which can aid in determining the depth of the virtual object [27].

### B. Feedback Design

The objective of this study was to investigate how different feedback modalities affect reaching movement strategy. To this end, we provided visual and haptic feedback. Terminal visual feedback was provided when the ball representing the user's hand was in contact with the target ball in the VR space. The default color of the target ball was yellow, but the color of the target ball became red when it come in contact with the user object.

Haptic feedback was implemented to provide the sensation of an object in the VR space. Because the robot was used as a haptic interface, impedance control [37] was exploited. It enabled us to provide compliant interaction in physical human-robot interactions (pHRI) [38]. In our proposed system, the desired pose was defined on the surface of the VR object. The deeper the contact with the object, the stronger the force feedback that was provided. We provided feedback on the sensations of the target ball and the virtual wall surrounding the participants' workspace using impedance control. In addition, the secondary torque was applied to maintain the robot's pose [39].

### C. EMG Measurement and Analysis Method

The study utilized surface EMG (sEMG) to measure the EMG activity in the participants' upper arms. Five muscles were selected for analysis: the biceps brachii, triceps brachii, upper trapezius, anterior deltoid, and posterior deltoid. The EMG signals were sampled at a frequency of 1 kHz and filtered using a Butterworth filter with cutoff frequencies of 20 Hz and 400 Hz. Additionally, a notch filter was used to remove the 50 Hz signal noise.

Peak latency was defined as the time between the appearance of the target ball and the peak value of the sEMG signal. The peak latency was obtained from the first movement segment, which was between the initial hand position and the first target position. Only the first target position was the same for comparison, and the rest of the target positions were randomized.

### D. Kinematic Variables

Five kinematic variables were selected: movement time, mean velocity, peak velocity, time-to-peak velocity, and velocity peak number. These variables were calculated as the mean of all 16 movement segments, eliminating the influence of path length variation. Each movement segment began when a new target ball appeared and ended when the target ball disappeared after contact with the participant's hand. Movement time was defined as the time required to complete one movement segment. The peak velocity was the maximum absolute velocity recorded during each movement segment. Mean velocity was calculated as the average reaching velocity of each movement segment. The time to peak was expressed as the time from the appearance of the new target to the time the peak velocity was recorded. This measure distinguishes the reaching movement between “ballistic phase” and “correction phase” [40]. The “ballistic phase” movement begins from the moment when visually triggered until the peak velocity is reached, and the “correction phase” movement requires more precision to touch the target. Movement smoothness was measured by counting the velocity peak number in a movement segment. To define the peak velocity, the velocity profile was first scanned for the local minima and maxima. When the difference between the minimum and the next maximum exceeded the cut-off limit of 20 mm/s, it was counted as a velocity peak. In addition, the time between two subsequent peaks had to be at least 150 ms. These variables were based on [22] and [41].

### E. Exercise Protocol

Ten healthy, right-handed participants were recruited (male:7, female:3). All participants were neuromuscularly intact. The participants were between the ages of 20 and 42 years (mean $27.8\,\pm {\,7.4}$ years). The procedures were approved by the Institutional Committee for Medical Ethics, and all participants provided written informed consent.

Each participant performed all measurements on the same day. They were allowed to practice the reaching task for approximately 1 min to familiarize themselves with the task. We ensured that the participants had a clear understanding of the task requirements through verbal confirmation before the measurement. Measurements were conducted according to the protocol presented in Table 3. We developed this protocol based on the work presented in [19]. Our modifications to the original design aim to reduce the number of repetitions by taking into account the potential effects of muscle fatigue [42]. With these considerations, this adapted protocol can be effectively utilized for future measurements of stroke patients, providing a practical and efficient solution for clinical settings. In this reaching task, two target ball sizes were prepared: a small-ball (radius: 84 mm) and a big-ball that was twice the size of the small-ball. During baseline and retention, participants could see the target object and their hand location on the screen (concurrent visual feedback) without any terminal feedback. However, in the training phase, concurrent feedback and terminal feedback were provided. Three types of feedback were provided: visual, haptic, and combined visual and haptic (multi-feedback) modalities. There was no time constraint for the task, but it took approximately 60 s to complete eight repetitions of the reaching exercise. After completing the experimental protocol with the big-ball mode, all participants rested for one minute before starting the exercise with the small-ball mode to avoid any arm muscle fatigue.

**TABLE 3:** Exercise Protocol

Feedback	Exercise	Repetitions
Concurrent visual	Baseline	16
Concurrent visual + terminal visual	Training	24
Concurrent visual	Retention	16
Concurrent visual + terminal haptic	Training	24
Concurrent visual	Retention	16
Concurrent visual + terminal multimodal	Training	24
Concurrent visual	Retention	16

The exercise had no time constraints.

### F. Statistical Analysis

A two-way repeated measures ANOVA test was performed to examine statistical differences in the effect of feedback modalities and object size (two factors) on kinematic variables and sEMG peak latency. Sphericity was checked using Mauchly's sphericity test. Statistical differences were tested using post-hoc t-tests with Bonferroni corrections. Statistical analyses were performed using the MATLAB R2022 software. The level of significance was set at $p < . 05$.
